# GrassSV – hybrid method to detect structural variants in high throughput DNA-seq data

**DOI:** 10.1371/journal.pcbi.1014406

**Published:** 2026-06-22

**Authors:** Dominik Witczak, Krzysztof Sychla, Julia Wysocka, Artur Laskowski, Wojciech Frohmberg, Marta Glowacka, Alicja Dzik, Piotr Lukasiak, Jacek Blazewicz, Aleksandra Swiercz

**Affiliations:** 1 Institute of Computing Science, Poznan University of Technology, Poznan, Poland; 2 ECBiG, European Center of Bioinformatics and Genomics, Poznan University of Technology, Poznan, Poland; 3 Institute of Bioorganic Chemistry, Polish Academy of Sciences, Poznan, Poland; CANADA

## Abstract

Genetic diversity is crucial for populations to adapt and survive in dynamic environments. This diversity arises from genetic mutations, which manifest in the genome as structural variants (SVs). Several types of SVs exist, but not all are equally easy to detect. Current SV detection tools tend to specialize in certain SV types or require the use of multiple tools to obtain a comprehensive variant profile, which increases computational cost and complexity. While some methods excel at identifying breakpoints, they often struggle with accurately classifying variant types, and their precision depends strongly on data quality and sequencing technology. At present, the majority of available genomic data originates from high-quality short reads, which remain the most affordable sequencing technology. In this manuscript, we introduce GrassSV, a novel and computationally efficient method that employs a hybrid pattern-matching approach to detect all major classes of structural variants using short-read sequencing data. GrassSV integrates depth-of-coverage analysis with contig-based pattern recognition to ensure both sensitivity and precision while minimizing false positives and runtime. Its robustness was demonstrated on the human Genome in a Bottle dataset, as well as on synthetic data derived from the yeast genome, where it achieved high accuracy across all SV types at a lower computational cost compared to existing methods. This makes GrassSV a practical alternative to multi-tool pipelines typically required for comprehensive SV detection. GrassSV is available at https://github.com/Domomod/GrassSV under GPL-3.0 license and the benchmark at: https://github.com/Domomod/GrassBenchmark.

## Author summary

Structural variants (SVs) are large genomic alterations that can profoundly influence gene function, regulation, and phenotype. Despite their biological importance, accurately detecting SVs from sequencing data remains a major computational challenge. Existing tools are often optimized for specific types of variants or rely on multiple algorithms to achieve full coverage, which increases computational cost and complexity. In this study, we present GrassSV, a hybrid approach for structural variant detection using short-read sequencing data. Our method combines coverage-based analysis with pattern recognition from assembled contigs, enabling comprehensive identification of deletions, insertions, inversions, duplications, and translocations within a single pipeline. We developed GrassSV to provide researchers with a practical, accurate, and efficient tool for large-scale genome analyses. We evaluated GrassSV on both synthetic and real datasets, showing that it detects all major SV types with high precision while reducing false positives and runtime compared to existing methods. By balancing accuracy and efficiency, GrassSV offers a cost-effective solution for genomic research and supports ongoing efforts to understand genetic variability in human and model organism populations.

## Introduction

Genetic variations encompass a wide range of sizes, from single nucleotide alterations to insertions or deletions of dozens of nucleotides (INDELs) and even rearrangements of larger DNA fragments. While single nucleotide variants (SNVs) occur on average once every 1,000 nucleotides (0.1%) when comparing two humans, structural variants (SVs) occur at a rate of 1.5% and represent a major type of variation among individuals [1,2]. Certain regions, such as telomeres or centromeres, are particularly prone to the appearance of SVs.

Structural variants commonly occur in deletions or duplications, known as copy number variations (CNV), but can also include inversions, translocations, and insertions. Events over 50–1,000 bp (depending on the publication) are typically classified as SVs. [3,4]. When SVs occur within protein-coding regions, they can disrupt gene function and gene regulation [5,6]. Additionally, SVs can affect the 3D organization of the genome and influence interactions between genes, or between genes and their regulatory elements [7,8].

Complex SVs, which are combinations of multiple structural variants located in the same region, have been reported, for example, in Mendelian disorders [5,9]. Moreover, SVs play a significant role in cancer development by amplifying oncogenes or deleting tumour suppressor genes [10].

Detecting SVs is essential in a clinical settings for personalized medicine, where understanding a patient’s unique genetic makeup can support inform diagnosis, treatment plans, and prognostic predictions. Despite these advances, challenges remain in accurately detecting and interpreting SVs due to their complexity and the limitations of current technologies. Finding variations in genomes approaches can be classified into four groups [2,11]. The first three described below involve first mapping reads to the reference genome and then detecting variants based on clues specific to the approach. In contrast, the last approach tries to assemble the genome *de novo*, and then looks for differences between the genomes by comparison with the reference.

- Read Depth (RD) analysis – this approach detects copy number variation by examining the depth of coverage across the genome. Variations in RD indicate regions of deletions and duplications. The methods working in RD usually cannot precisely detect breakpoints of variants.
- Pair-End mapping (PE) – SVs are identified by analyzing the distances and orientation between paired-end reads. Any deviations from the expected distance and orientation allow for the detection of a wide range of variants.
- Split Read mapping (SR) – this approach detects SVs by aligning reads that span breakpoints of structural variants. These reads cannot be mapped continuously to the genome but can be split into two parts and precisely indicate SV breakpoints.
- Assembly-based approach – these involve assembling the short reads into longer contigs, which can then be aligned to the reference genome to identify SVs. This method can resolve complex SVs that are difficult to detect using other approaches, for example, insertions that cannot be detected by mapping to the reference genome.

Several tools have been proposed that utilize one or more of the aforementioned approaches for detecting SVs. Cortex, for example, uses a colored de Bruijn graph to detect SVs and other shorter rearrangements directly during the *de novo* assembly process across multiple genomes simultaneously [12]. Similarly, SGVar identifies variations during the assembly process, but instead of using a de Bruijn graph, it employs a string graph, where uniquely overlapping reads are merged into single sequences, forming nodes in the graph [13]. Other tools, like Assemblytics [14] and SMARTie-SV [15], rely on aligning the assembly to a reference genome to identify SVs.

Another group of tools first aligns the reads to a reference genome and then identifies SV candidates based on factors like coverage, uneven mapping, and the distance and orientation of paired-end reads. For example, CNVnator [16] focuses on detecting CNVs by analyzing read depth in windows of a given size. BreakDancer [17] detects SVs by analyzing the mapping results of a read and its paired-end read; regions with several abnormally mapped read pairs are classified as containing specific types of SVs.

To enhance the precision of breakpoint detection, split-read analysis is often employed. Pindel, for example, uses split-read mapping to identify breakpoints of large deletions and medium-size insertions [18]. Delly [19] combines both paired-end and split-read data to achieve high accuracy in predicting breakpoints, enabling it to detect small deletions effectively, though larger events can still be challenging to distinguish.

Some tools incorporate a third source of information—depth of coverage—to address issues with uneven or abnormal read mapping. For instance, Lumpy [20] and Manta [21] both use this approach. Manta is particularly versatile, capable of analyzing germline samples from multiple individuals or performing paired analyses of normal and tumor tissues. GRIDSS, on the other hand, excels in detecting breakpoints by operating on a positional de Bruijn graph-based assembler, providing very precise results [22].

Advancements in sequencing technologies, such as long-read sequencing and high-throughput methods, along with sophisticated computational tools, have significantly improved the detection and characterization of SVs. These technologies allow for more accurate and comprehensive mapping of SVs, which is crucial for understanding their biological impact. The detection of SVs has led to significant breakthroughs in genomics and biomedical research, uncovering new genetic mechanisms underlying various diseases and traits. However, there remains a need for more robust and efficient tools, especially those capable of handling the complexity and diversity of SVs.

In this context, we introduce GrassSV, a new program designed to detect SVs in the genome with higher precision and recall. GrassSV addresses the limitations of existing tools and offers a comprehensive solution for SV detection, enhancing both research and clinical applications. The program identifies potential SV breakpoints and performs *de novo* assembly of reads in these regions. Through in-depth analysis of contig mapping patterns, GrassSV can accurately annotate the specific SVs present, even in case of more complex variants, like translocations or duplications.

To test the usefulness of our algorithm, we performed tests on synthetic data that could be verified with measures of precision and recall and on the golden standard benchmark dataset from Genome in a Bottle Consortium (GIAB). We selected sample NA12878 (HG001) and Illumina short-reads libraries with coverage 30X. The GIAB dataset was widely used to generate the benchmark of different kind of genetic variation: SNVs, INDELs, and larger variations within complex and repetitive fragments of genome like segmental duplications or major histocompatibility complex (MHC) [23].

## Design and implementation

The GrassSV pipeline aims to reduce the cost of DNA assembly by leveraging the fact that circa 99% of our genetic makeup is identical across all individuals. This means that most of the genetic code does not provide useful information for detecting structural variants. To take advantage of this, GrassSV employs a hybrid approach, performing depth of coverage analysis before *de novo* assembly. Analyzing depth of coverage is a well-known method often used to detect structural variants, as their occurrence in the genome affects the read coverage when aligned to the reference genome. This analysis pinpoints potential sites of genetic variation, allowing for the extraction of reads with the highest informational value. Further assembly of these reads produces long contigs, enabling the precise detection of SV types and breakpoint locations. Notably, this approach results in significant computational savings, as *de novo* assembly is performed on a subset of reads much smaller than the initial set.

### GrassSV – pipeline overview

**Fig 1:**
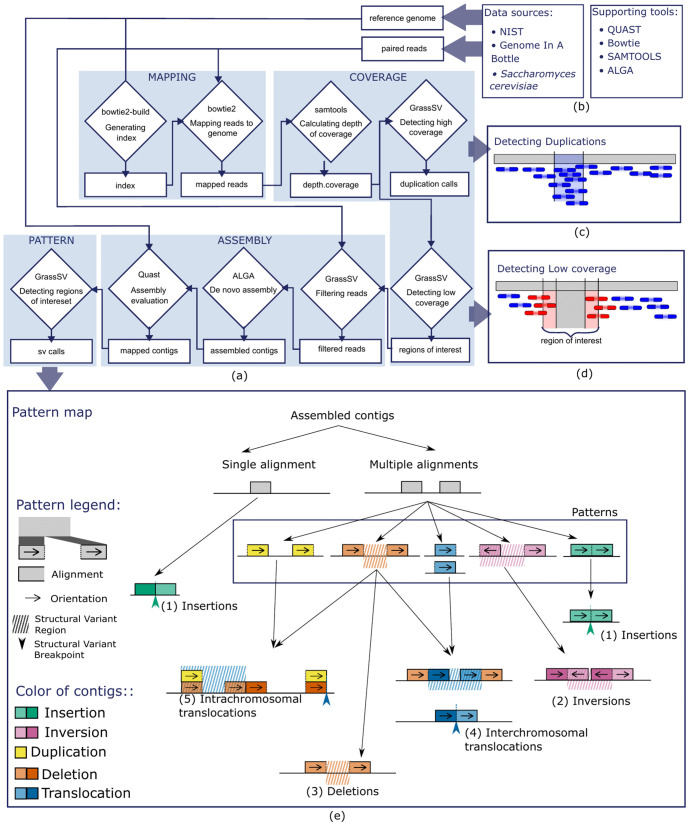
Structural variants detection using GrassSV method. **(a)** Overview of GrassSV steps and their input/output data. **(b)** External fundamentals of GrassSV. **(c)** Duplications are detected mainly by observation of higher coverage. **(d)** Regions with lower coverage of reads are potential places of other structural variants. **(e)** Exploration of patterns over alignments manifesting given structural variant types.

GrassSV is modular and customizable, with four core components facilitating efficient SV detection (Fig 1).

#### Mapping module.

GrassSV begins by preparing the NGS reads for downstream processing in its Mapping Module. Here, GrassSV uses Bowtie2 [24] for indexing and mapping reads into the SAM format for reference genome alignment, with options to replace it with other SAM-compliant tools.

#### Coverage analysis module.

The Coverage Analysis Module aggregates mapped reads to evaluate genome continuity and identify regions with atypical coverage. Coverage depth is calculated for each genomic position using samtools [25]. Next, regions showing significantly higher or lower coverage than the genome-wide average are flagged as Regions of Interest (ROIs). These ROIs indicate potential structural variant breakpoints where genomic continuity may be altered. Higher coverage regions are interpreted as potential duplication events, and are analyzed separately, whereas regions with reduced coverage are associated with deletions or other types of structural variation. Reads mapping within or around the identified ROIs—extended by a configurable margin—are selected for subsequent analysis steps. The parameters defining coverage thresholds (default 10) and ROI margins (150 bp) are user-adjustable, allowing flexible adaptation of the method to various datasets and sequencing depths.

#### Assembly module.

GrassSV then processes the ROI-specific reads, together with reads that did not map to the reference genome, in the Assembly Module. We perform *de novo* assembly using ALGA tool [26]. In this module, we can easily exchange with tools such as Velvet [27] or GRASShopPER [28] to accommodate different assembly preferences. The resulting contigs from this assembly process offer unbiased information about the sequenced genome and enhance GrassSV’s capability to detect insertions or other complex SVs.

#### Pattern recognition module.

GrassSV’s Pattern Recognition Module maps contigs to the reference genome using QUAST tool [29] to identify SVs through discontinuous sequence mapping. A dedicated algorithm detects deletions, duplications, inversions, insertions, and translocations using color-coded pattern recognition. We note that duplication-like mapping patterns can appear in intrachromosomal translocations; nonetheless, actual duplications are detected only based on increased read coverage within the Coverage Analysis Module. Figure 1e presents a pattern map illustrating the pattern recognition process.

GrassSV categorizes potential detected mutations through an analysis of the relative position and relationship of mapped contigs. The algorithm begins by identifying ’simple patterns’, which are then used to detect different SV types. Five colors represent the patterns characteristic of each SV type. For example, red contig fragments that map entirely on both sides of a variant, with the same mapping orientation, indicate a deletion. A similar blue pattern, which also indicates increased coverage in the area, suggests a duplication. A yellow pattern, where contig fragments map in reverse orientation, indicates an inversion. Typically, such a region exhibits two inversion patterns on both sides of the breakpoint. Detecting translocations, both interchromosomal (between chromosomes) and intrachromosomal (within the same chromosome), is more complex. A translocation involves two SV events: the deletion of a fragment from one location and its insertion in another. Therefore, it’s common to see a combination of translocation and deletion or duplication and deletion patterns. However, insertions, which involve the insertion of additional fragments not present in the reference genome, are the most challenging to detect accurately. They cannot be reliably identified using coverage depth analysis because these require the presence of the fragment in the reference. Also, the paired reads approach could possibly detect the presence of insertion by looking for the cluster of the one-end of the paired read mapped, but the inserted sequence would not be detected. Hence, only hybrid tools or those employing *de novo* assembly approaches can potentially detect the occurrence of insertion and the inserted sequence itself.

### Synthetic Dataset

To evaluate the performance of the algorithm, we prepared synthetic datasets based on the *Saccharomyces cerevisiae* (common yeast) reference genome, introducing predefined structural variants of various types and sizes (500–5000 bp). Basing on the modified FASTQ genome files, we generated five datasets with synthetic NGS reads with the ART tool [30]. The average sequencing coverage was set to 30×, following previous studies [31] that examined the impact of coverage depth on computational time and SV detection accuracy. Each dataset contains 500 random structural variants, either of a single type, (e.g., only deletions – DEL_500, duplications – DUP_500, insertions – INS_500, and inversions – INV_500,) or a mixture of all variant types (100 of each; SV_ALL).

For the synthetic data, the sites of the introduced mutations were recorded in .bed format, enabling precise validation of the reported variants. With the synthetic data, each variant call could be classified as either a True Positive or a False Positive. Additionally, undetected mutations could be classified as False Negatives. This systematic classification allowed for a comprehensive evaluation of each program’s accuracy and reliability.

Each program generates variant calls that include information about the type of variant and its coordinates (breakpoints). However, these calls may not always be entirely accurate, which must be considered in the analysis. In this study, two approaches were proposed to detect deviations from the ground truth.

The first approach assumes that a “True Positive” is a variant call that contains all mutation breakpoints, overlap with the original annotated SV and correctly classifies the variant type (see: Region-based validation).

The second approach models “True Positives” as breakpoints detected within a breakpoint margin from the generated mutation breakpoints (see: Breakpoint-based validation).

#### Region-based validation.

Each reported SV is discriminated based on the intersection with the ground-truth SV, compared to the union of the two:


$$\begin{array}{c} \frac{Length(SV_{reported}\cap SV_{true})}{Length(SV_{reported}\cup SV_{true})} \end{array}$$

Reported SVs are considered correct if the **region overlap score** exceeds a specified threshold, calculated as the percentage of intersection over union between the reported and true SV regions.

#### Breakpoint-based validation.

Breakpoints are specific locations within the genome where a given SV is detected. Two SVs are compared based on these locations, with a specified **breakpoint margin** represented by the number of base pairs. Unlike region-based validation, only the breakpoints are compared, and the classification of the SV type is not considered.

### GIAB Dataset

We utilized a dataset from GIAB consortium, that aims to develop reference materials and methods for genome sequencing and variant calling. Seven individuals were sequenced using a variety of technologies, including Illumina, 10X Genomics, BioNano optical maps, Nanopore, and PacBio [32].

As a benchmark in our project, we used the NA12878_HG001/ NISTv4.2.1/GRCh38 dataset with the following NGS runs: “SRR2052337,” “SRR2052338,” “SRR2052339,” “SRR2052351,” and “SRR2052352.” The resulting depth of coverage for the dataset is equal to 30X.

We worked with a pre-prepared file containing all annotated SNPs, INDELs, and SVs. Out of almost 8.4 million annotations, we selected only those with a length greater than 50 bp, representing SVs. This selection resulted in more than 27,000 breakpoints, and we categorized each SV as either a deletion or an insertion, depending on whether the altered fragment was shorter (deletion) or longer (insertion). However, it is possible that other types of SVs are also present in the dataset, potentially as combinations of more than one variant type.

## Results

Basing on recent articles with comparison and evaluation of SV detection tools [31,33,34], we select five methods representing different approaches for finding SV. GRIDSS [22], Manta [21], Lumpy [20], Delly [19], Pindel [18], and our method GrassSV were evaluated against both synthetic and real (GIAB) data. Synthetic datasets were used to assess the accuracy of the pipeline, allowing for validation under controlled conditions, while the real dataset was employed to test the toolchain’s performance in analyzing human genomes and its practical application.

The results for the synthetic datasets can be directly compared with the ground truth since SVs were deliberately introduced during their preparation, with their exact positions known down to the nucleotide. In contrast, real datasets contain a set of known SVs, which may not be exhaustive, as they were detected using software on actual data and not intentionally introduced.

### Synthetic Data

**Fig 2:**
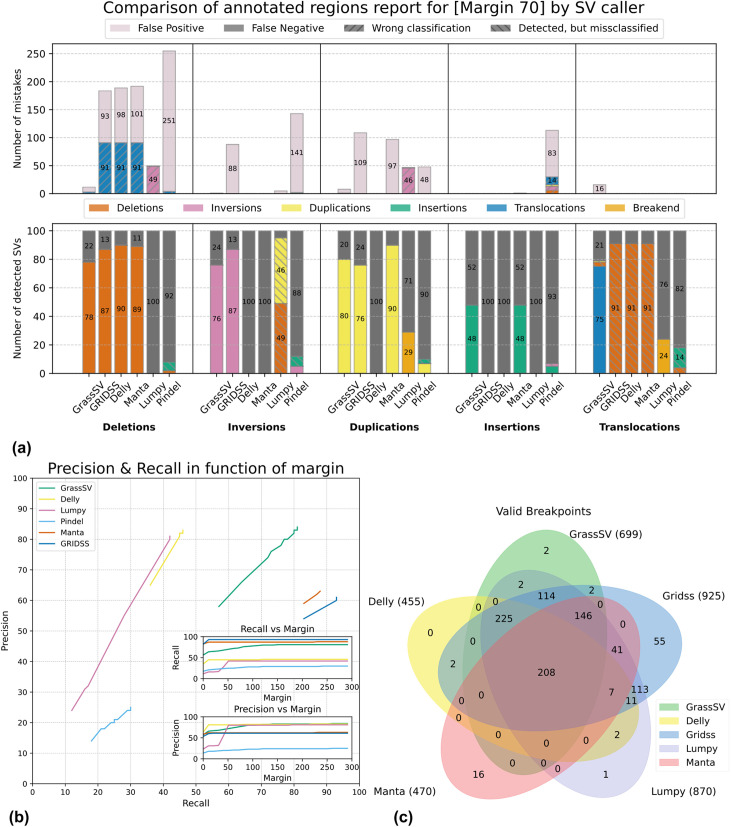
Results for the synthetic data benchmark SV_ALL. **(a)** This plot illustrates how each method performs in relation to different types of SVs (DEL, INV, DUP, INS, and TRN). The lower panel shows the number of detected variants, with a maximum of 100 for each SV type. The dark grey bars indicate undetected variants (false negatives). The upper panel displays misclassified SVs and SVs detected at loci where no variant is present (false positives, FP). For example, GrassSV correctly detected 76 DELs (lower panel) but misclassified a few (<5) TRNs as DELs and also detected 9 additional DELs, indicated as FPs (upper panel). Lumpy, on the other hand, detected 30 DELs, classified as BreakEnd (lower panel), but misclassified 49 INV as DELs (upper panel). Additionally, misclassified SVs can be seen in the lower panel, such as a few (<5) DELs in the TRN section for GrassSV and 49 DELs in the INV section for Lumpy. **(b)** Precision and Recall of each method, defined as a function of margin. The *breakpoint margin* represents the tolerance for locating breakpoints. The smaller panels show how Recall and Precision change as the margin around breakpoints varies. **(c)** Venn diagram comparing the robustness of GrassSV to other methods in detecting valid breakpoints.

The analysis of the synthetic dataset was performed using both region-based and breakpoint-based validation methods. Figure 2 presents the results for the SV_ALL dataset, which includes all SV types: deletions (DELs), duplications (DUPs), inversions (INVs), insertions (INSs), and translocations (TRNs). In the upper part of Fig 2a, the methods are evaluated based on the correct annotation of SV types, separated into different columns. We considered only the annotated SVs for which the region overlap score exceeds 70%. The lower figures (Fig 2b, 2c) focus solely on precision in detecting breakpoints (duplicated breakpoints were discarded).

Figure 2 demonstrates that the performance of each method strongly depends on the SV type. Several methods were able to detect deletions (DELs), including GrassSV, GRIDSS, Manta, and Delly. GrassSV and GRIDSS correctly detected inversions (INVs) and duplications (DUPs), whereas Manta identified only duplications. In contrast, Pindel performed poorly in most cases and produced a high number of false-positive calls. A more detailed analysis revealed that Pindel was primarily effective for short SVs up to 1 kbp. GrassSV and Manta were the only methods capable of reliably detecting insertions (INSs).

In some cases, methods detected SVs but misclassified them; for instance, GRIDSS, Delly, and Manta detected translocations (TRNs) but annotated them as duplications (DUPs), while Lumpy detected most inversions but classified them as deletions or duplications. In other cases, Lumpy failed to assign variants to a specific category, reporting them as break ends (BNDs). The highest number of false positives was introduced by Pindel, GRIDSS, and Manta. Notably, most tools generated considerably more false DELs compared to other SV types.

We further analyzed precision and recall for each method as a function of breakpoint margin (Fig 2b), selecting a 70 bp breakpoint margin as a reference point, since all methods performed reasonably well at this threshold. GRIDSS detected the largest number of SVs but exhibited reduced precision, while Lumpy, Delly, and Pindel introduced numerous errors and still missed a substantial number of valid SVs. GrassSV achieved the best balance between precision and recall.

Figure 2c presents the precision of each method in detecting breakpoints. Most breakpoints were identified by more than one method, whereas Lumpy, Delly, and Pindel detected fewer than half of all true breakpoints (Pindel was excluded from the chart for clarity). GRIDSS detected the majority of correct breakpoints, including many unique ones, but also produced a large number of invalid breakpoints.

**Fig 3:**
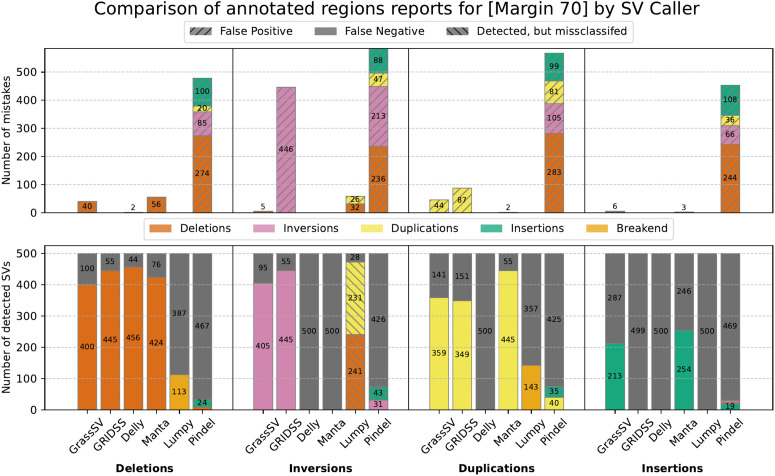
Results for synthetic data benchmark DEL_500, INV_500, DUP_500, and INS_500. Each dataset contains only one type of introduced variation. The lower panel shows the number of detected variants, where the maximum number is 500. Correctly detected and classified variants are marked in solid colors, with the color described in the legend. Misclassified variants are denoted with stripped color, and the dark grey color indicates undetected variants (false negatives). The upper panel shows false positives (FPs), and the color indicates the type of variant that was wrongly detected.

Similar trends were observed in Fig 3, where each dataset contained only a single SV type. GrassSV was able to detect and correctly annotate all SV types, whereas the other methods failed to identify at least one variant category. Both GRIDSS and Manta successfully detected DELs and DUPs, with the former also identifying a high number of INVs and the latter showing good performance for INSs. Delly detected mostly DELs. Lumpy struggled with SV type annotation, while Pindel detected most variants with a region overlap score above the 70% threshold but also produced a high number of false positives.

### GIAB Data

The real data used courtesy of “The Genome in a Bottle Consortium,” provides some inside how analyzed algorithms perform in realistic scenarios. It is important to note that such datasets are not perfect, as not all SVs are detected with complete accuracy, but they represent the best approximation available. For this experiment, only GRIDSS and GrassSV were compared, as these two algorithms performed reasonably well on the synthetic datasets.

**Fig 4:**
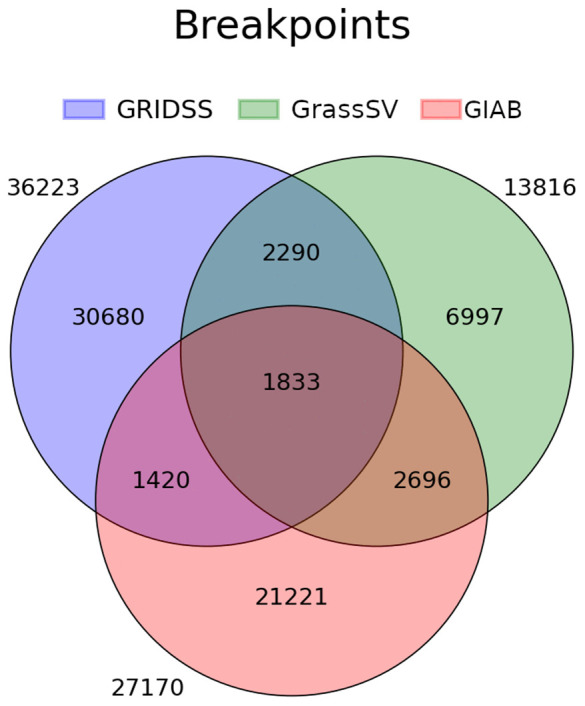
Valid breakpoints in GIAB dataset. Venn diagram showing the number of breakpoints detected by each method and reported in GIAB benchmark. Those variants detected only by single method are considered misclassified according to GIAB benchmark.

In Fig 4, we can see that GRIDSS and GrassSV detected 3,253 and 4,529 correct SVs, respectively (according to the GIAB benchmark). Neither method detected 21,221 SVs, and both detected some SVs that are not part of the benchmark (9,287 for GrassSV and 32,970 for GRIDSS). Although neither method performed perfectly, GRIDSS detected around 30% fewer correct SVs and nearly three times more SVs that are not part of the benchmark compared to GrassSV. Based on the synthetic data experiment, we expected GRIDSS to generate a significant number of false positives, which suggests that GrassSV performed more reliably on the real dataset.

The GIAB dataset was processed in parallel batches. The first step—common to both methods—involved building the genome index and mapping the reads, which took 63 minutes. The total computation time for GRIDSS was about 9.5 hours, while for GrassSV it was approximately 5.5 hours. GrassSV reached a maximum memory usage of 250 GB.

Computational tests for large datasets must be executed on high-performance computing infrastructure, where tasks can be parallelized and sufficient memory resources are available. For the GIAB dataset, analyses were performed on an HPC system with Intel Xeon Platinum 8268 CPUs and BIGMEM configuration. For smaller datasets, such as yeast, GrassSV can be efficiently run on a standard workstation (e.g., Intel i7-11700K, 32 GB RAM).

## Discussion

The results obtained from both synthetic and real datasets provide valuable insights into the performance of the analyzed SV detection algorithms. GrassSV and GRIDSS were the most promising tools in this study, as they demonstrated reasonable accuracy in detecting various types of SVs under controlled (synthetic) and realistic (real-world) conditions. GrassSV consistently outperformed GRIDSS, particularly in terms of correctly identifying INSs in synthetic data and reducing the number of false positives in the GIAB dataset.

The synthetic data evaluation highlighted that GrassSV achieved a good balance between precision and recall, making it an effective tool for detecting SVs in regions with known mutations. GRIDSS, although robust in detecting DELs and DUPs, struggled with INSs and generated a significant number of false positives, which was confirmed by its performance on the real dataset. Notably, GRIDSS was less accurate in SV annotation for the synthetic dataset—though many breakpoints were correctly identified, not all SVs were reported, and some were duplicated, leading to an increase in false positives. In contrast, Manta and Delly were more conservative, producing fewer false positives but also missing a higher proportion of true variants. In the GIAB data analysis, GrassSV detected a higher number of correct SVs while minimizing false positives compared to GRIDSS, showing that it is more reliable for real-world genome analysis. Additionally, GrassSV completed the analysis 20% faster than GRIDSS, further emphasizing its efficiency.

When compared to Pindel and Lumpy, GrassSV also demonstrated clear advantages. Both Pindel and Lumpy had significant issues with misclassifying SV types and detecting breakpoints with high precision, particularly in complex variants like insertions and inversions. Pindel, in particular, introduced a high number of false positives in the synthetic data analysis, while Lumpy struggled with correctly annotating SVs, often labeling them as Break Ends instead of specific variant types. Similarly, Manta tended to overcall duplications, while Delly detected only deletions and translocations. This underscores GrassSV’s superior ability to accurately detect and classify SVs across a broad spectrum of variant types while maintaining balanced performance and computational efficiency.

It is important to note that no single SV detection tool performs optimally across all variant types and datasets. Each method has its own strengths and limitations, depending on factors such as variant size, sequencing depth, and genomic context. While combining multiple SV detection tools is a common strategy to increase confidence in variant calls, it also introduces additional complexity in result integration and interpretation. In this context, GrassSV offers an alternative approach by enabling the detection of all major SV types within a single, unified pipeline, reducing the need for extensive multi-tool workflows. Thus, rather than replacing existing methods, GrassSV can simplify or complement current practices, particularly in scenarios where consistency of variant annotation and workflow efficiency are important.

## Availability and future directions

GrassSV is available as an open-source tool at: https://github.com/Domomod/GrassSV under the GPL-3.0 license. GrassSV can be run using Snakemake, a workflow engine that allows you to easily run an entire pipeline [35,36]. If the previous run of the program did not complete, the program can continue computing based on the previously completed steps

The benchmark dataset used in this study can be accessed at: https://github.com/Domomod/GrassBenchmark.

Despite the emergence of long-read sequencing technologies, short-read next-generation sequencing (NGS) remains widely used and continues to dominate public genomic databases. This is due to the broad accessibility of short-read platforms and the extensive legacy data generated over the past decade. As such, there remains a critical need for accurate and efficient SV detection tools tailored to short-read data. GrassSV addresses this need by providing robust structural variant detection with balanced precision, recall, and runtime.

Looking ahead, the underlying pattern-matching methodology of GrassSV could be adapted to long-read sequencing technologies. However, this approach cannot be applied directly. Instead, a long-read–specific version of the pipeline would likely start with the analysis of mapped reads (e.g., a sorted BAM file), similar to tools such as CuteSV [37] and Sniffles2 [38]. Given the lower uniformity of long-read coverage, identifying ROIs based solely on depth would be less reliable; a better strategy would involve using alignment operations from CIGAR strings. Both CuteSV and Sniffles2 rely on split-read alignments to classify SV types and apply clustering and refinement procedures to consolidate candidate variants.

The underlying geometric logic of these methods aligns with the conceptual framework implemented in GrassSV. As long-read sequencing becomes increasingly accessible, adapting GrassSV to this data type may further improve both the sensitivity and specificity of SV detection. While certain features—such as the hybrid use of alignment and coverage depth—may not directly carry over, the pattern-recognition approach could still prove valuable, especially for detecting complex rearrangements or handling low-coverage datasets.

In summary, GrassSV provides a competitive, efficient, and versatile framework for structural variant detection in short-read data, while also offering a promising foundation for future adaptation to long-read sequencing workflows.
